# Visible near infrared reflectance molecular chemical imaging of human *ex vivo* carcinomas and murine *in vivo* carcinomas

**DOI:** 10.1117/1.JBO.25.2.026003

**Published:** 2020-02-24

**Authors:** Shona Stewart, Marlena Darr, Heather Gomer, Aaron Smith, Arash Samiei, James Christopher Post, Ralph J. Miller, John Lyne, Jeffrey Cohen, Patrick J. Treado

**Affiliations:** a ChemImage Corporation, Pittsburgh, Pennsylvania, United States; b Allegheny General Hospital, Pittsburgh, Pennsylvania, United States

**Keywords:** molecular chemical imaging, hyperspectral imaging, surgery, kidney cancer, breast cancer, lung cancer, renal cancer, intraoperative imaging

## Abstract

**Significance**: A key risk faced by oncological surgeons continues to be complete removal of tumor. Currently, there is no intraoperative imaging device to detect kidney tumors during excision.

**Aim**: We are evaluating molecular chemical imaging (MCI) as a technology for real-time tumor detection and margin assessment during tumor removal surgeries.

**Approach**: In exploratory studies, we evaluate visible near infrared (Vis-NIR) MCI for differentiating tumor from adjacent tissue in *ex vivo* human kidney specimens, and in anaesthetized mice with breast or lung tumor xenografts. Differentiation of tumor from nontumor tissues is made possible with diffuse reflectance spectroscopic signatures and hyperspectral imaging technology. Tumor detection is achieved by score image generation to localize the tumor, followed by application of computer vision algorithms to define tumor border.

**Results**: Performance of a partial least squares discriminant analysis (PLS-DA) model for kidney tumor in a 22-patient study is 0.96 for area under the receiver operating characteristic curve. A PLS-DA model for *in vivo* breast and lung tumor xenografts performs with 100% sensitivity, 83% specificity, and 89% accuracy.

**Conclusion**: Detection of cancer in surgically resected human kidney tissues is demonstrated *ex vivo* with Vis-NIR MCI, and *in vivo* on mice with breast or lung xenografts.

## 1 Introduction

Kidney cancer is among the 10 most common cancers in both men and women.^1^ Overall, the lifetime risk for developing kidney cancer is about 1 in 48 for men or 1 in 83 for women.^2^ Renal cell carcinoma (RCC) is responsible for 80% to 85% of all primary renal neoplasms.^3^ In the United States, there are approximately 74,000 new cases and almost 15,000 deaths from RCC each year,^1^ and combined data from the National Program of Cancer Registries and Surveillance, Epidemiology, and End Results Program show a 4.3% annual increase in the incidence of localized RCC from 2001 to 2010.^4^

For many cancers for which the cancerous tumor is localized to a specific area of the body, tumor removal, also known as curative or primary surgery, is the primary treatment.^5^^,^^6^ During this surgery, the tumor and some of the nearby healthy tissues are removed. The tissue around the excised tumor is called the margin. The current standard of treatment for a small renal mass is partial nephrectomy (PN, removal of a portion of the kidney) when technically feasible or radical nephrectomy (RN, removal of the entire kidney) for large tumors.^7^ The objective during PN is to remove the tumor completely while preserving normal renal parenchyma (NRP) and obtaining a negative surgical margin (NSM), i.e., the outer edge of the tissue excised from the kidney is free from tumor cells.^8^ While positive surgical margins (PSMs) in PN and RN, i.e., tumor cells remaining at the outer edge of excised tumor tissue, are rare (0% to 10%),^8^^–^^14^ they have been associated with RCC recurrence and worsened patient outcomes.^8^^,^^13^^,^^15^^,^^16^ Complete tumor removal with NSM is dependent on a surgeon’s ability to identify all of the tumor tissues and cells. Currently, there is no intraoperative imaging device to detect kidney tumors during excision. Intraoperative ultrasonography has long been used as an intraoperative imaging technique for tumor localization and delineation during PN, but because the probe stays in contact with the tissue, it is not practical for real-time guidance during resection, and only a few studies report on the intraoperative assessment of surgical margins within the United States.^17^^–^^20^ For these studies, ultrasonography performed intraoperatively during PN identified NSMs with 100% sensitivity and 97% to 100% specificity when compared with the final histopathological examination.^17^^–^^20^ While near-infrared (NIR) fluorescence is useful for assessing the surgical cavity and assessing tissue perfusion, the technology has limited tissue penetration depth and requires contrast agents, which can be problematic due to patient intolerance and impact on surgical workflows.^21^^–^^24^ Augmented reality, in which preoperative or intraoperative images are superimposed onto the surgical field, has shown some success differentiating between tumor and NRP. However, the lack of a viable solution to soft tissue deformation due to surgical dissection, bleeding, and tumor distortion represents a major limitation to applying augmented reality during tumor resection.^25^^,^^26^ One prospective technology for real-time intraoperative tumor detection and margin evaluation is molecular chemical imaging (MCI). MCI is a noncontact optical imaging modality, which can be performed noninvasively, quickly, and without the use of contrast agents. We have demonstrated, in feasibility studies, MCI detection of anatomic structures, including blood vessels, nerves, and cervical lymph nodes, in the presence of obscuration in both *ex vivo* and *in vivo* models.^27^ We are now developing an MCI-based intraoperative imaging device for improving accurate tumor localization during tumor removal surgery. The function of this device is to provide to the surgeon enhanced detection images highlighting tumor cells in a surgical field of view in real time (at least as fast as 10 frames per second) during resection, facilitating complete removal of the tumor while obtaining an NSM and preserving as much healthy tissue as practical. While we report in this paper results primarily observed in a human kidney cancer model, we anticipate this technology to be applicable to many localized tumors for which curative surgery is the principal treatment.

### 1.1 Molecular Chemical Imaging for Advance Surgical Visualization

MCI, also known as hyperspectral imaging, has the potential to transform intraoperative tumor detection and margin assessment. We use the term MCI when referring to hyperspectral imaging applied to medicine and biomedicine to reflect the measurement of molecular content key to addressing medical needs. MCI integrates digital imaging with spectroscopy to provide both spatial and molecular information about a sample. Rather than visualizing an object with only red, green, and blue (RGB) wavelengths, as seen by the human eye, MCI utilizes up to hundreds of wavelengths to yield additional information about a sample, such as molecular content.^28^ With this high volume of information, MCI offers the possibility of identifying and distinguishing components that may not be obvious to the human eye. Each tissue type, critical anatomic structure, and tumor has an intrinsic molecular composition that can be measured by MCI as a unique molecular signature or spectral biomarker. The spectral biomarkers, if reproducible, form the basis of real-time tumor detection and localization when empowered by machine learning and computer vision algorithms. A number of spectroscopic and hyperspectral technologies, including fluorescence spectroscopy, diffuse reflectance spectroscopy, and Raman spectroscopy, have been successfully implemented to study cancers of various origins, including breast cancer,^29^^,^^30^ melanoma,^31^ lung cancer,^32^ head and neck cancers,^33^^,^^34^ liver cancer,^35^ and renal cancers.^36^^–^^38^ While these studies demonstrate tumor differentiation and in some cases tumor visualization, the methodologies are limited to evaluating biopsies or probing excised tumors or the tumor bed after excision. The MCI strategy is to provide continuous real-time images showing tumor localization and augmenting the surgeon’s ability to determine the location of an NSM during excision.

We report in this paper the results of two feasibility studies performed to determine if real-time MCI-based tumor detection shows promise and to provide insight into design of an MCI-based intraoperative imaging device. (1) First, we evaluate MCI-based tumor detection in human *ex vivo* kidney specimens excised from patients diagnosed with kidney cancer. Molecular chemical images of kidney specimens comprise spectra corresponding to tumor and nontumor tissues. We confirm that tumor and nontumor spectra are distinct, and from the hypercubes, we create kidney tumor score images. Tumor score images are single-frame images in which the pixels corresponding to tumor are brighter than the pixels corresponding to the surrounding, nontumor tissue. Kidney tumor score images are generated in two different ways: a best-in-class method employing a multivariate statistical model and taking into account a large spectral range, and a second approach, which requires only two wavelength image frames. The statistical method for generating score images is the more robust method and provides an indication of method feasibility. The two-wavelength approach is less robust but shows promise for real-time application. Development and implementation of computer vision algorithms to the score images generates detection images, which define the locations of pixels identified as tumor. Detection images are evaluated in terms of sensitivity, specificity, and accuracy, based on the ground truth of tumor location as indicated by the surgeon during MCI data collection. (2) Second, we assess the possibility of *in vivo* MCI tumor detection by performing MCI on anaesthetized mice inoculated with patient-derived tumor xenografts. We show that *in vivo* spectra of tumor and nontumor tissues from six inoculated and four cancer-free mice can be differentiated, even when obscured with skin. In addition, we generate representative tumor score and detection images.

## 2 Materials and Methods

### 2.1 Samples

#### 2.1.1 *Ex vivo* human kidneys

A total of 22 human kidney specimens with tumors were obtained postoperatively at Allegheny General Hospital (Allegheny Health Network, Pittsburgh, Pennsylvania) with institutional review board (IRB) approval and with each patient’s informed consent. Specimens were removed from patients via laparoscopic or open nephrectomy (radical or partial) as part of their standard of care due to the presence of cancerous tumors. Specimens comprised tumor and surrounding nontumor tissues such as NRP, renal fat, and renal sinus fat (RSF). Fourteen of the cancers were confirmed as clear cell RCC, two were papillary RCC, one was a chromophobe RCC, two were transitional cell carcinomas (TCCs), and three were mixed carcinomas.

Upon surgical removal, each specimen was immediately transported to the surgical pathology laboratory. The urological surgeon provided ground truth for each kidney specimen with visual inspection and palpation, identifying the locations of the tumor and the other tissues of interest. Visible near-infrared (Vis-NIR) MCI hypercubes were collected from entire specimens. For most specimens, more than one hypercube was required to capture the entire sample. After imaging, specimens were then prepared for histological examination by pathology technicians as per standard of care. The cancer diagnosis was confirmed by a pathologist via histopathology.

#### 2.1.2 *In vivo* mouse models

*In vivo* experiments were performed on Non-Obese Diabetic Severe Combined Immune Deficiency gamma female mice purchased from the Jackson Laboratory (Bar Harbor, Maine). In total, MCI hypercubes were collected from 12 mice. To induce breast cancer growth, three mice were subcutaneously inoculated 6 weeks previously with patient-derived invasive ductal carcinoma (IDC) breast tumor in the right flank region. At the same time, three tumor-negative mice were not inoculated but otherwise cared for over the same period of time. To induce lung cancer growth, three additional mice were subcutaneously inoculated 6 weeks prior to imaging with patient-derived lung adenocarcinoma tumor in the right flank region. At the same time, three additional tumor-negative mice were not inoculated but otherwise cared for over the same period of time. Two different tumor models were imaged because of the lack of availability of six mice with the same tumor.

During imaging experiments, mice were anesthetized by intraperitoneal injection of a ketamine/xylazine/acepromazine mixture. For each experiment, a pair of mice was imaged together: one mouse with tumor, paired with one tumor-free mouse. Six MCI hypercubes were collected from mice as follows.

• Mouse pair 1: Breast cancer model, intact and with fur (“intact” MCI hypercube).
• Mouse pair 2: Breast cancer model, intact with fur around location of tumor on both mice shaved off (“intact and shaved” MCI hypercube).
• Mouse pair 3: Breast cancer model, right flank region opened to expose the inner skin flap and the abdomen on the mouse body. In the mouse with cancer, the tumor is on the skin flap (“exposed” MCI hypercube).
• Mouse pair 4: Lung cancer model, intact and with fur (“intact” MCI hypercube).
• Mouse pair 5: Lung cancer model, intact with fur around location of tumor on both mice shaved off (“intact and shaved” MCI hypercube).
• Mouse pair 6: Lung cancer model, right flank region opened to expose the inner skin flap and the abdomen on the mouse body. In the mouse with cancer, the tumor is on the skin flap (“exposed” MCI hypercube).

Following completion of MCI, mice were sacrificed by either cervical dislocation or thoracotomy and submitted for necropsy examination to evaluate pathology of tumors and associated tissues via hematoxylin and eosin staining. A pathologist confirmed the presence, types, and locations of the tumors after excising them.

### 2.2 Phenomenology Selection

There are a number of viable phenomenologies that have potential applicability to MCI and advanced surgical visualization. These include diffuse reflectance methods in the Vis-NIR and shortwave infrared (SWIR) spectral regions, fluorescence emission, and Raman scattering. Raman spectroscopy, the most specific of these phenomenologies, has been used to evaluate a significant number of biological tissues and cells in many applications.^39^ However, Raman scattering is an infrequent phenomenon, and the methodology has low sensitivity. Therefore, it cannot be performed at real-time image frame rates. Fluorescence imaging is more sensitive than Raman scattering and has already been accepted in the medical industry. However, the fluorescent dye to be ingested can induce an allergic reaction in the patient. The diffuse reflectance spectroscopic methods have been widely recognized in the evaluation of biological systems.^40^^,^^41^ They have the advantage of being nondestructive and noncontact, requiring no sample preparation, having high analytical sensitivity, and being very fast, allowing real-time sensing.^42^ One of the main benefits of Vis-NIR spectroscopy is to exploit measurement of oxygenated and deoxygenated hemoglobin, thus indicating the viability of tissue.^41^^,^^43^ At longer wavelengths, peaks characteristic of lipids and water provide information about the presence of edema, burns, and tumors.^44^^,^^45^ Limitations are typically associated with reduced specificity and susceptibility to blood obscuration. SWIR spectroscopy makes use of prominent absorption peaks of water, lipids, and collagen,^41^ and in this spectral region, susceptibility to blood obscuration is decreased. However, SWIR technology has been relatively limited because InGaAs sensor arrays, frequently used as SWIR detectors, have been cost-prohibitive compared to detectors compatible with Vis-NIR spectroscopy. In addition, wide distribution of InGaAs sensors has been somewhat limited by some U.S. Defense-related policies, such as International Traffic in Arms Regulations.^41^ Although both Vis-NIR and SWIR methodologies have comparable favorability for intraoperative imaging, we selected Vis-NIR for three reasons: (1) higher sensitivity than Raman, (2) higher specificity than fluorescence, and (3) lower cost focal plane array than SWIR.

### 2.3 Molecular Chemical Imaging Devices

**Fig. 1:**
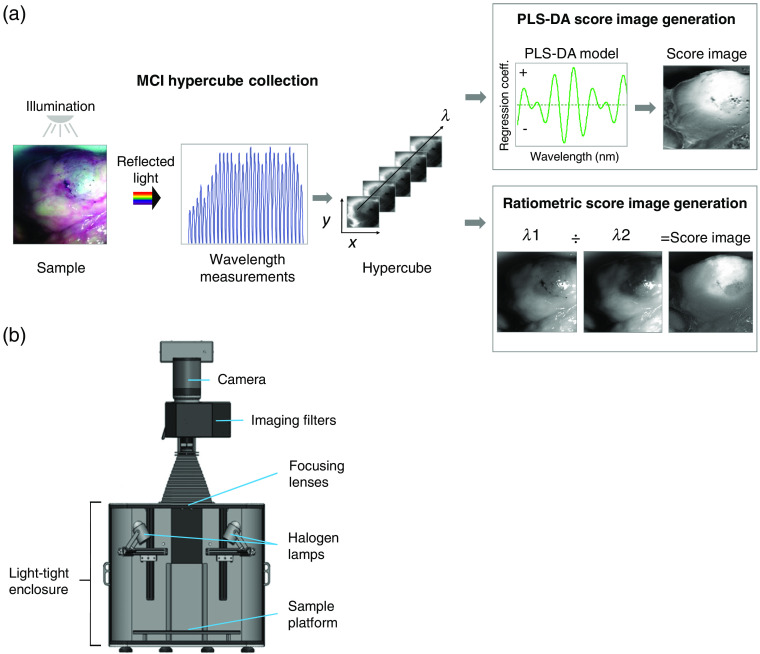
MCI. (a) Vis-NIR MCI wavelength-resolved images are collected over the free spectral range (400 to 1100 nm) and assembled into a hypercube comprising three dimensions: x and y spatial dimensions and λ spectral dimension. Each pixel in an MCI hypercube is a Vis-NIR reflectance spectrum, which is evaluated using multivariate algorithms or a ratiometric approach to generate target tissue score images. (b) Schematic of a representative wide-field hyperspectral imaging system and its components.

The methodology implemented for collecting and processing molecular chemical images is outlined in Fig. 1(a). A molecular chemical image (also known as an MCI hypercube) comprises a three-dimensional data set in which each image frame represents reflectance intensities at a specific wavelength. MCI hypercubes are acquired using a wide-field hyperspectral imaging system such as that shown in Fig. 1(b). Samples are placed on the sample platform and illuminated with halogen lamps (Ushio America, Inc., Cypress, California), which generate broadband white light covering the range 400 to 1100 nm. Light reflected from the sample is captured by zoom and focusing lenses and directed through a liquid crystal tunable filter (LCTF, Perkin Elmer, Waltham, Massachusetts). The LCTF is tuned electro-optically to allow discrete wavelengths of light to pass to a charge-coupled device (CCD). As they are detected on the CCD, the filtered wavelengths form a two-dimensional wavelength-resolved image frame comprising reflectance intensities from every pixel on the CCD. The final stack of wavelength-specific image frames is the MCI hypercube, with three dimensions: x and y spatial dimensions and wavelength λ. Each pixel in the hypercube contains a spectrum that is determined by the molecular composition of the tissue in the sampled field of view at the pixel location.

MCI hypercubes of *ex vivo* human kidney specimens were collected from 400 to 1100 nm in steps of 5 nm (141 frames). The camera utilized for these experiments was a CCD (Princeton Instruments, New Jersey). The spatial resolution was 0.80 mm, and field of view size was 100  mm×100  mm. For specimens larger than 100  mm×100  mm, multiple hypercubes were collected in order to image all parts of the sample. Multiple hypercubes representing the same specimen from different perspectives are called sample fields of view in this report. Specimens were imaged intact. If specimens were cut in half to observe the interior of the tumor (as part of the patient’s standard of care), MCI hypercubes were acquired from the tumor interior as well.

*In vivo* hypercubes of breast and lung cancer mouse models were collected over the spectral range of 400 to 1100 nm in steps of 5 nm. Detection was achieved using a CCD (Teledyne Photometrics, Tuscon, Arizona), and spatial resolution over a 163  mm×124  mm field of view was 0.40 mm.

### 2.4 Image Preprocessing

Hypercubes collected from *ex vivo* and *in vivo* models were corrected to account for instrument response. Instrument response correction was performed by dividing raw sample hypercubes by a hypercube collected from a 99% reflectance standard (Labsphere, North Sutton, New Hampshire). Corrected hypercubes were converted from reflectance (R) to absorbance (A) using the equation A=log(1/R).

Image spectra were extracted from each tissue type in corrected MCI hypercubes. Spectral extraction was achieved by selecting a group of pixels, known as a region of interest (ROI) in a hypercube, which corresponded to tissue type as annotated at the time of data collection. Each spectrum is the average of the individual pixel spectra within the ROI selected. For each tissue type in each field of view, 10 ROIs were selected for analysis. All extracted spectra were vector-normalized. Spectra representing specific tissue types are termed signatures in this report. Signatures of renal tumor, NRP, fat, and RSF were extracted from corrected human kidney hypercubes. Signatures extracted from intact, intact and shaved, and exposed murine models represent tumor (IDC and lung adenocarcinoma), muscle and subcutis from tumor-positive mice, and muscle and subcutis from tumor-free mice.

### 2.5 Analysis

#### 2.5.1 Signature analysis

Signature analysis was performed on the kidney specimen data to identify spectral similarities and differences between tissue types in the *ex vivo* models. Signature spectra were subjected to a multiclass partial least squares discriminant analysis (PLS-DA) calculation to yield dendrograms and misclassification rates of the tissue types.

PLS-DA represents a best-in-class analysis method, typically for two-class systems. It is a well-known multivariate statistical tool for supervised data classification, model generation, and performing data reduction. In addition, PLS-DA models are often used to predict class membership (or classification) of unknown measurements. PLS-DA is described in more detail elsewhere.^46^^,^^47^ In our multiclass PLS-DA, a “one-vs-all” approach is used. Using this technique, k classifiers (where k = the number of tissue types) are built, in which each tissue type is the target class and all other classes are combined into the negative class. The k classifiers are applied to each sample, and the sample is predicted to belong to the class, or tissue type, with the highest score.

The signature analysis was performed on 440 fat spectra from 44 fields of view, 530 NRP spectra from 53 fields of view, 120 RSF spectra from 12 fields of view, and 440 tumor spectra from 44 fields of view over the spectral region of 520 to 1050 nm (107 wavelengths). PLS-DA classifiers were built using 7 factors, and leave-one-field-of-view-out cross validation was performed, in which all spectra for one tissue type field of view were left out as the test class.

#### 2.5.2 Tumor discrimination

**Fig. 2:**
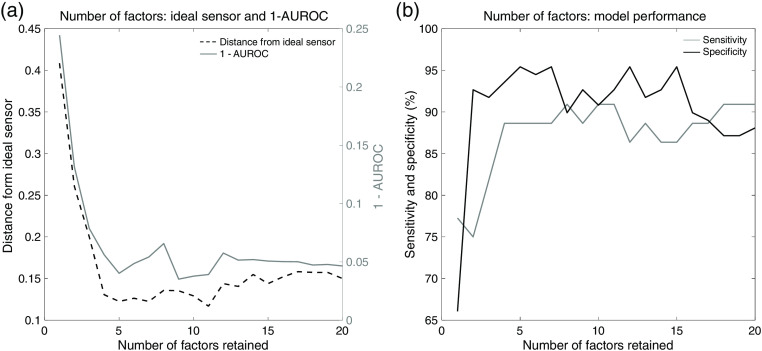
Evaluation and selection of factors retained for PLS-DA. (a) Plots illustrating distance from an ideal sensor (i.e., 100% sensitivity, 100% specificity on the ROC curve) and 1–AUROC with number of factors retained. (b) Plots showing sensitivity and specificity exhibited by the model with number of factors retained.

Tumor discrimination was demonstrated by generating two-class PLS-DA models for the *ex vivo* human kidney cancer study and for the *in vivo* human-derived tumor xenografts in mice. In these models, tumor was the positive class, and all other tissues were considered the negative class. Selecting the minimal number of factors for producing optimal model performance is a critical step when generating models. Selecting too few factors may yield a model that does not account for a majority of the population variance. Selecting too many factors can result in overfitting the model, thus modeling noise instead of true variance. In this study, two plots were used as guidance when selecting the number of factors for a specific model. The PLS-DA model is built repeatedly using cross validation, varying the number of factors retained to generate the two plots. Figures 2(a) and 2(b) illustrate the effect of number of factors on test performance. Figure 2(a) exhibits the impact of a number of factors on the area under the receiver operating characteristic curve (AUROC) and distance from the ideal sensor [i.e., ideal receiver operating characteristic (ROC) curve performance–100% sensitivity and 100% specificity]. Both 1–AUROC (solid line) and distance from ideal sensor (dashed line) are more favorable when lower. In this plot, we look for the elbow at which the slope of both curves tapers off. We choose the number of factors close to this elbow (to reduce the chance of overfitting the data) and that reflects low metric values. In this case, we determined the most appropriate number of factors to be 5. Figure 2(b) is a plot showing the sensitivity (gray line) and specificity (black line) of the test as number of factors changes. Assuming that high values for both metrics is desirable, we can see that the most favorable sensitivity and specificity combination (approximately 88% and 96%, respectively) is obtained when 5 factors are retained. Retaining 8 factors may increase sensitivity to 90%. However, this comes with a drop in specificity and AUROC. Therefore, we used 5 factors to build this model.

##### *Ex vivo* human kidneys

A PLS-DA model for renal tumor was generated from 440 renal tumor spectra from 44 fields of view and 1090 nontumor spectra (comprising fat, NRP, and RSF) from 53 fields of view over the spectral region of 520 to 1050 nm (107 wavelengths). Five factors were used, and leave-one-field-of-view-out cross validation was performed.

##### *In vivo* mouse models

Lung cancer and breast cancer spectra from 10 mice in total were combined to build a PLS-DA model for tumor versus nontumor. The two intact tumor-free mice were not included in this model because spectra due to fur only were omitted. The model was generated from 60 tumor spectra from the intact, the intact and shaved, and the exposed tumor-positive mice (three breast cancer mice + three lung cancer mice), and from 120 nontumor tissue spectra extracted from the 10 tumor-positive and cancer-free mice. Ten spectra represented each tissue included in the model. Using the methodology described, we selected 8 factors and built the model over the spectral range of 520 to 1050 nm. Leave-one-tissue-out cross validation was performed.

Evaluation of a PLS-DA model is based on the model’s ability to discriminate the positive class from the negative class. Metrics used to assess model performance were sensitivity, specificity, accuracy, and AUROC. The ROC curve is a plot of sensitivity versus 1-specificity of a test for a binary classification system, where a perfect AUROC value is 1.000.^48^ A ROC curve is generated by sweeping a threshold value across the PLS-DA score values or probabilities produced by the model. The optimal threshold value, which is the value maximizing sensitivity and specificity, is also determined during this process.

#### 2.5.3 Score image generation

Corrected MCI hypercubes were analyzed to generate score images, which are high-contrast, single-frame images that highlight target tissue (i.e., tumor) against background tissues. Score images are the basis of enhanced visualization of target tissues using MCI. In these studies, we generated score images using two approaches: a multivariate approach and a ratiometric approach. Because the former approach utilizes a larger volume of spectral data, we anticipate the statistical model to explain a representative amount of the variance and therefore yield more representative score images. We apply the multivariate approach to provide a robust evaluation of feasibility of study success. The ratiometric approach can be implemented in real time and so is a favorable methodology to explore.

##### Multivariate approach

PLS-DA score images are generated by applying a PLS-DA model to specimen MCI hypercubes. In a PLS-DA score image, a higher pixel intensity corresponds to a higher score in the model, correlating with the positive class in the model (in these studies, tumor). Negative class members (i.e., nontumor tissues) have lower scores in the PLS-DA model and thus exhibit lower pixel intensities. This way, contrast is created between regions classified as tumor and regions classified as nontumor.

PLS-DA score images were generated for eight of the *ex vivo* human kidney specimens.

##### Ratiometric approach

A ratiometric score image is generated by dividing one hypercube frame within an MCI hypercube by another. Wavelength image frames are selected by comparing signature spectra of the target and background. To maximize target/background contrast, the target should exhibit lower absorption than the background at the numerator wavelength and higher absorption than the background at the denominator wavelength. For individual, simple tissue specimens, a wavelength pair can be determined manually. However, for complex tissue specimens and for wavelength pairs that generate score images across several specimens, we use a software program developed in-house (Spectral Chef™, ChemImage, Corp., Pittsburgh, Pennsylvania). Ratiometric score images were generated for the human kidney tumor specimens and the murine tumor models.

#### 2.5.4 Score image evaluation

PLS-DA and ratiometric score images are assessed by the magnitude of the contrast between target and background pixels (signal-to-noise-ratio, SNR) and AUROC. SNR is calculated as the mean of the target pixel intensities minus the mean of the background tissue pixel intensities, divided by the standard deviation of the background tissue pixel intensities. Higher SNR and AUROC values will help yield a more inclusive detection.

#### 2.5.5 Detection image generation

Detection images are created from the score images using the computer vision and image processing steps outlined below.

1. Spectral noise is reduced and image contrast is increased with one or more of the following: glare removal, noise removal, image normalization, and image enhancement.
2. The target is detected, using image segmentation methods. A global threshold method is initially applied to extract the foreground object (the brighter pixels), followed by the application of cascade filters to suppress false positives. Subsequently, active contour segmentation^49^ is used to obtain a more precise target boundary.
3. For the purpose of visualization, the RGB image and MCI score image are registered by maximizing their mutual information.^50^ After registration, the detection is colorized and overlaid onto the RGB image.

#### 2.5.6 Detection evaluation

Detection images are evaluated with several metrics: sensitivity, specificity, and accuracy are standard performance metrics. Also determined is the Jaccard index, or Intersection over Union (IOU). This figure of merit is calculated by dividing the intersecting areas of the detection and ground truth by the total combined areas of detection and ground truth.^51^ This tool takes into account both false negatives and false positives. A higher IOU indicates a better match between target detection and ground truth.

## 3 Results and Discussion

### 3.1 Signature Analysis

Successful detection of tumors and other critical structures with MCI is dependent on exploitation of diffuse reflectance spectroscopy and will be limited by any lack of discrimination specificity between target tissues and background tissues. Signature analysis is undertaken to assess differences between tissue types using the signature spectra extracted from MCI hypercubes.

**Fig. 3:**
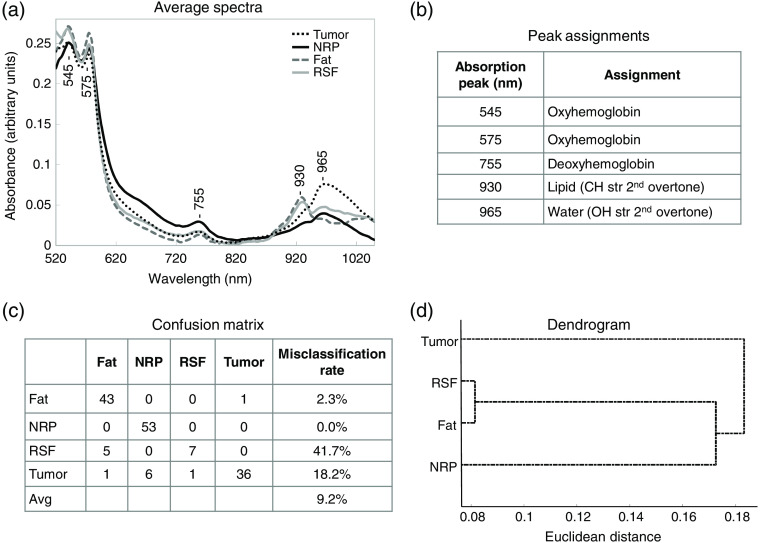
Signature analysis of human kidney tissue. (a) Average absorption spectra representing tumor, NRP, fat, and RSF from 22 human kidney specimens. (b) Corresponding spectral peak assignments. (c) Confusion matrix, showing classifying ability of multiclass signature model. Misclassification rate indicates the percentage of out-of-class classifications. (d) PLS dendrogram, which shows the hierarchical relationship between the classes.

The average absorption spectra of renal tumor (dashed black line), NRP (solid black line), fat (dashed gray line), and RSF (solid gray line) are shown in Fig. 3(a). There are distinct differences between the representative spectra, particularly between 850 and 1050 nm. Absorption peaks correspond to molecular vibrations occurring as a result of light absorption, scattering, and reflection and can be assigned to specific molecular or functional groups present in the tissue. The macromolecules and functional groups contributing to the absorption peaks in Fig. 3(a) are outlined in Fig. 3(b).

The intense peaks at 545 and 575 nm correspond to hemoglobin, in particular oxygenated hemoglobin. Those peaks frequently dominate the visible spectrum of biological tissues. The companion peak at 755 nm denotes the additional presence of deoxygenated hemoglobin in these samples.

The other two primary peaks at 930 and 965 nm reflect the presence of lipids and water, respectively. The components with the highest lipid peaks are the fat (found on the exterior of the kidney) and the RSF (found within the renal sinus of the interior of the kidney). The spectral peaks in this region have different relative intensities depending on tissue type. Notable is the peak due to the OH stretch of water at 965 nm, which is highest in intensity for tumor. This may be explained by the structure and composition of the majority of the tumors sampled in this study. Clear cell RCCs typically have high water content and are highly vascular.^52^ Although papillary and chromophobe RCCs are typically hypovascular,^53^ they comprise a small fraction of the tumor population and therefore would not significantly influence the spectral peak due to water content.

A dendrogram, such as that shown in Fig. 3(d), illustrates the hierarchical relationship between classes. Classes that are connected with the shortest lines are the most similar. In this PLS model, the RSF and fat are the most similar, seen by the very short lines connecting the two classes. RSF and fat spectral similarity is also supported in the confusion matrix [Fig. 3(c)] in which RSF misclassifies as fat nearly 42% of the time. That both fat and RSF are composed of lipid and exhibit very similar reflectance spectra explains the high misclassification rate. The dendrogram also shows that, in this model, NRP is more similar to the fatty components than tumor is to the fatty components. The notable misclassification of tumor as NRP 13% of the time may be rationalized by the imprecise tumor annotations made on the imaged bulk tissue. Tumor borders were not, in this study, confirmed at microscopic dimensions with the histopathology findings, so we expect a small amount of unintended physical overlap of tumor and NRP. Future studies will include carefully designed quantitative histopathological (microscopic) assessment of tumor edges as the reference methodology so that MCI tumor margins may be evaluated more precisely.

Signature analysis gives us an indication of anticipated discrimination performance when Vis-NIR MCI is employed as a tissue visualization modality. While signature analysis is a predictor of tissue discrimination performance, there are several inherent limitations, including (1) multiple tumor signatures, including from highly vascular and hypovascular tumors, have been grouped into one class, which may mask differences between these subtypes; (2) spatial accuracy of ground truth at the tumor border is limited; (3) other tissue types present in the fields of view that were not annotated may degrade the purity of the signatures. Despite these limitations, moderate-to-high discrimination performance of kidney tumors is indicated.

### 3.2 *Ex Vivo* Human Renal Tumor Discrimination

**Fig. 4:**
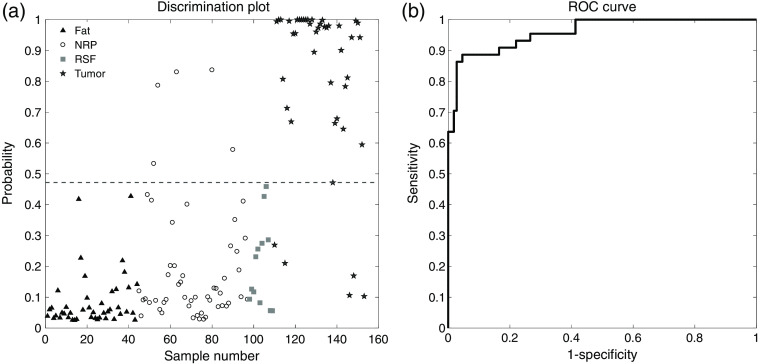
PLS-DA model for human renal tumor. (a) Discrimination plot showing PLS-DA classification of tissue types. At the optimal probability threshold of 0.47, the accuracy of this model is 94%, the sensitivity is 89%, and the specificity is 95%. (b) ROC curve, with an integrated area of 0.960, illustrates the significant discrimination power of this model.

Results of a PLS-DA model for renal tumor are shown in the discrimination plot and ROC curve in Figs. 4(a) and 4(b), respectively. The discrimination plot illustrates classification of the tumor (solid stars), RSF (solid squares), NRP (circles), and fat (solid triangles) data. Data classified as tumor fall above the threshold line (dashed line), and data classified as nontumor fall below the threshold line. The renal tumor model performs very well, with 93.5% accuracy, 88.6% sensitivity, and 95.4% specificity for renal tumor. The high discrimination performance is also illustrated in the ROC curve, which has an AUROC of 0.960. Correctly classified specimens include tumor obscured by a thin (∼1  mm) layer of fat. The tumor specimens misclassified as nontumor in the model do not appear to adhere to a particular pattern or stratify by the tumor types, which comprise clear cell RCC, papillary RCC, chromophobe RCC, TCC, and mixed carcinomas. All of the nontumor spectra, which are misclassified as tumor, correspond to NRP.

### 3.3 Renal Tumor Visualization and Detection

Score images provide enhanced visualization of target tissues. Tumor score image contrast is dependent on the underlying spectral differences between tumor and nontumor tissues. The subsequent computer vision-derived detection images are created from the score images and provide definition to the tumor tissue borders. Automated detection is the final step in MCI-based enhanced visualization of target tissues. Ideally, a detection methodology for a specific target is developed over a substantial sample population and applied automatically to new score images. To visualize the detection images, they are pseudocolored and overlaid onto the corresponding RGB images for location context. In a surgical environment, an MCI device would generate scores and detection image overlays in real time as a video, and hence fast data acquisition and processing is vital. PLS-DA, while best-in-class for supervised classification of spectral data, typically involves the capture of images at many discrete wavelengths, and as a result may not be readily performed in real time. An alternative method for generating tumor tissue score images is to ratiometrically combine the wavelength frames. Score images created this way can be captured at substantially higher frame rates because only two individual wavelength-resolved images need to be collected, and the resulting score images can often exhibit appreciable contrast, even comparable to PLS-DA score images.

**Fig. 5:**
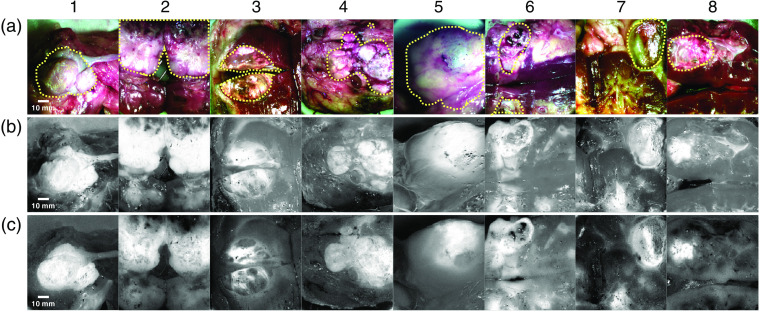
MCI score images of human renal tumors *ex vivo*. (a) RGB images, generated from MCI hypercubes. (b) PLS-DA score images generated by applying the PLS-DA model to representative MCI hypercubes. Higher pixel intensity indicates a higher probability of tumor. (c) Ratiometric score images. Contrast of tumor pixels (higher intensity) against darker background pixels is created with the ratio of two optimal wavelength images: 915/1000 nm. Performance metrics for each score image are shown in Table 1.

Representative PLS-DA and ratiometric score images for renal tumor are shown in Fig. 5. Figure 5(a) comprises RGB images (synthetic images generated from MCI hypercubes) with tumor annotations in yellow. Figure 5(b) gives corresponding PLS-DA score images, generated by applying the PLS-DA model to MCI hypercubes, and Fig. 5(c) comprises score images generated ratiometrically. These images were generated by dividing the 915-nm frame by the 1000-nm frame of the specimen hypercubes. The ratiometric wavelength pair was determined using the principles outlined in Sec. 2.5.3 and averaging the NRP, fat, and RSF spectra to represent nontumor. This was determined for several kidney specimens simultaneously and is known as a “consistent case” wavelength pair. When developed over a complete and representative sample population, consistent case wavelength pairs are applied to any applicable specimen to generate a relevant score image.

**Table 1:** Performance metrics for kidney tumor score images (SNR and AUROC) and detections (IOU, sensitivity, specificity, and accuracy.)

	PLS-DA		Ratiometric					
	SNR	AUROC	SNR	AUROC	IOU	Sensitivity	Specificity	Accuracy
Avg. ± Std. Dev.	2.02±0.33	0.91±0.04	2.63±0.96	0.89±0.08	0.61±0.21	69%±21%	97%±4%	86%±12%

Score image and detection image performance metrics are displayed in Table 1. SNR and AUROC are used to evaluate score image quality, and IOU, sensitivity, specificity, and accuracy are utilized to assess detection performance. Score images generated by PLS-DA and the ratiometric method perform similarly, within standard deviation. Average AUROC of PLS-DA score images is slightly higher than for ratiometric score images and the converse is true for SNR. This suggests that for some biological systems, the ratiometric methodology can be an appropriate method for generating score images in real time without degrading target tissue contrast.

**Fig. 6:**
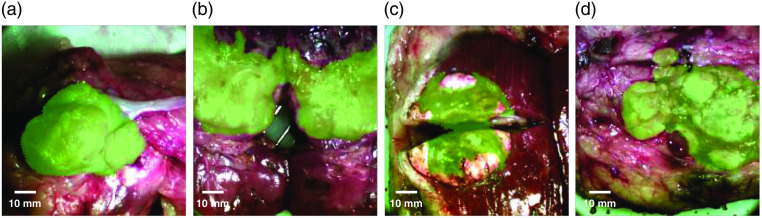
MCI detection images. Detections were created by applying computer vision algorithms to the ratiometric score images and overlaying the resulting detection onto the RGB image. Panels (a)–(d) correspond to score images in columns 1 to 4, respectively, in Fig. 5. Average performance metrics are shown in Table 1.

Detection images are generated using the pixel intensities in score images and a number of computer vision algorithms developed for intraoperative imaging applications. Figure 6 shows a subset of the calculated detections, generated from the ratiometric score images shown in Fig. 5, overlaid in green onto the corresponding RGB images. The associated metrics for detections, also listed in Table 1, show mixed results. On average, specificity and accuracy are high. Average IOU and sensitivity are somewhat lower, however, with a large standard deviation. Of the detections shown in Fig. 6, samples 1 and 4 exhibit high performance over all metrics: 0.94 IOU, 98% sensitivity, 99% specificity, and 99% accuracy for sample 1 and 0.85 IOU, 94% sensitivity, 96% specificity, and 95% accuracy for sample 4. For these samples, the detection captured most-to-all of the tumor and exhibited few false positives relative to the ground truth annotated in Fig. 5. Samples 2 and 3 exhibit high specificity (99%, 97% respectively) and accuracy (85%, 89% respectively), indicating few false positives. However, they have lower IOU (0.73, 0.58 respectively) and sensitivity (74%, 63% respectively), which indicate an omission of some of the tumor region. This is also reflected in the score images in Fig. 5(c), where we see high intensity pixels covering part but not all of the area denoted as tumors for samples 2 and 3.

Upon closer examination of the spectra from these tumors, we are able to hypothesize why these detections were less sensitive. Vis-NIR spectra from the lower and upper portions of the tumor in sample 2 are quite different. The spectra in the upper portion of the tumor, where the detection did not reach, indicate tissues that are higher in blood content than in the lower portion of the tumor. This is evident by the higher absorption oxyhemoglobin and deoxyhemoglobin spectral peaks in addition to higher water content, which is often associated with larger volume of blood. In addition, dark patches throughout the same region reflect tissue necrosis. The presence of necrosis and blood is a departure from the majority of tumor signatures sampled and so is not represented well by the ratiometric wavelengths selected for score generation. Further development of the methodology will include blood-obscured samples and necrotic tissue in training data sets to ensure that detection methodologies will yield score images accounting for the potential presence of blood and necrosis.

Similarly, the tumor in sample 3 is highly heterogeneous. The tumor regions not included in the detection in Fig. 6 comprise spectra that have a higher background than spectra from tumor regions included in the detection. In addition, the RGB image shows high reflection in those areas that would contribute to the higher background. Our objectives in future studies will include optimization of data collection methodology to include more heterogeneous tissues and to reduce specular reflection, in addition to improving glare reduction algorithm strategies.

The *ex vivo* studies provide proof of the concept that Vis-NIR MCI has a substantial potential for improving tumor visualization in renal cancers. To provide further evidence of successful implementation of MCI in intraoperative applications, and to begin to de-risk human *in vivo* imaging, we performed proof-of-concept *in vivo* measurements using mouse models.

### 3.4 *In Vivo* Tumor Detection

Our ultimate goal is to develop MCI for use *in vivo* in humans. To evaluate the feasibility of MCI detection of tumors *in vivo*, we have performed proof-of-concept experiments collecting MCI data from anaesthetized mice with and without tumor xenografts. The aim is to understand differences between live and dead tumors and to show in a limited data set that tumors were discriminated from nontumor tissues using MCI *in vivo* in live mice.

**Fig. 7:**
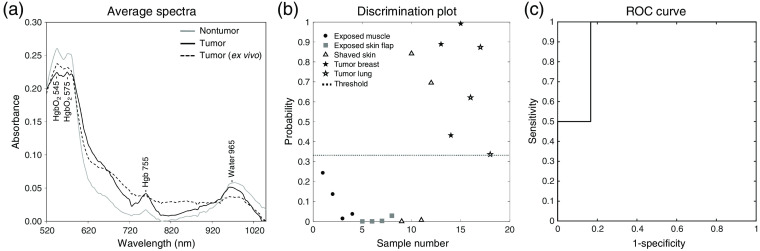
PLS-DA model for tumor *in vivo*. (a) Average absorption spectra representing *in vivo* and *ex vivo* tumor and *in vivo* nontumor tissues. (b) Discrimination plot illustrating classification of tumor and nontumor tissues (muscle, skin, and skin flap) from mice with and without tumor. (c) ROC curve illustrating performance of the model.

The average absorption spectra of tumor (IDC and lung cancers combined) are compared with nontumor tissues in Fig. 7(a) (solid black line and solid gray line, respectively). MCI was performed while mice were anesthetized, and to avoid the effects of motion on MCI data, hypercubes were collected at a faster rate than for *ex vivo* imaging. For this reason, the murine model spectra are slightly noisier. The absorption peaks dominating all spectra are those due to hemoglobin and water. The nontumor spectrum has the highest water peak at 965 nm, and oxygenated hemoglobin peaks at 545 and 575 nm, which is likely explained by the tissues contributing to this signal. Exposed muscle and shaved skin, in particular, are locations of high water content and vascularity. On the other hand, the tumor spectrum indicates a higher concentration of deoxygenated hemoglobin, with the peak at 755 nm and a broader envelope below 600 nm.

The discrimination plot and ROC curves of a PLS-DA model comprising *in vivo* breast and lung tumor tissue and nontumor tissues from three tumor-positive and three tumor-negative mice are shown in Figs. 7(b) and 7(c). Tumor data (IDC, solid black star; lung, white star) represents tumor measured through skin and fur, tumor measured through shaved skin, and exposed tumors on the skin flap. Nontumor data represent intact and shaved skin (triangle), exposed abdomen muscle (solid black circle), and exposed skin-flap tissue (solid gray square) from tumor-positive and tumor-negative mice. This model performs very well, with 100% sensitivity, 83.3% specificity, 88.9% accuracy, and an AUROC of 0.917. This favorable performance is likely, in part, because of the small test population, in addition to true spectral differences between the tumor and the nontumor tissues. The discrimination plot illustrates data classified as tumor (above the threshold, dashed line), and data classified as nontumor tissue (below the threshold). That all of the tumor data classify correctly indicates promise for MCI through obscurations since the tumor data include spectra from specimens exposed and under skin. Interestingly, the two nontumor data points that classify incorrectly consist of spectra extracted from the intact skin directly around the lumps created by both breast and lung tumor xenografts. This suggests the presence of tumor in a location that does not appear clearly to be tumor. Two possibilities for the misclassification are that the deformation of the intact skin (to accommodate the tumor mass) may cause some cross scattering of the reflected light from tumor and nearby nontumor tissues, or that the tumor cells do exist very close to the tumor mass in this case. The former phenomenon can be mitigated by building final tumor models with spectra from true tumor and true nontumor tissues and not from tissues that border tumor and nontumor.

**Fig. 8:**
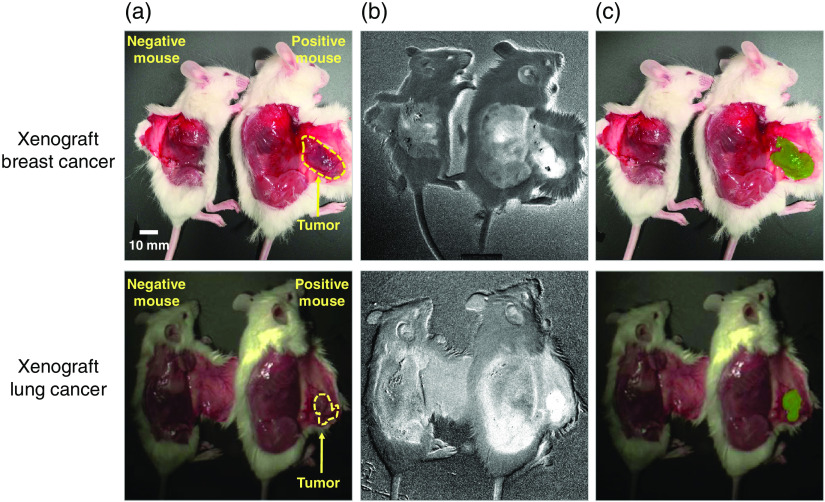
MCI score images of breast and lung cancer in mice *in vivo*. (a) Annotated RGB images of mice with (positive) and without (negative) xenografts. The tumors, located on the exposed skin flap (subcutis) near the flank, are annotated in yellow. (b) Ratiometric score images for lung cancer model (735/975) and breast cancer model (1035/625). (c) Tumor detections (in green) from ratiometric score images overlaid onto RGB images.

Because this *in vivo* exercise was performed to provide an indication of applicability of MCI to *in vivo*, real-time imaging, only ratiometric score images were calculated. These are shown in Fig. 8(b). Both breast cancer (1035/625 nm) and lung cancer (735/975 nm) score images and detection images show significant contrast between the tumors and the background tissues. SNR for IDC tumor is 6.6 and lung adenocarcinoma tumor is 4.7. Figure 8(c) yields moderate IOUs: 0.71 for breast tumor and 0.72 for the lung tumor. Because this is a limited data set, the detections generated are not assumed to be representative of a large population but serve only as examples of the application of *in vivo* MCI data to living subjects.

Ideally, method development, particularly ratiometric method development for real-time automated tumor detections, would be possible with the use of *ex vivo* samples. We have observed that *ex vivo* human tissues can provide considerable insight into optically targeting tumor and implementing strategies to yield advanced visualization in score images of tumor-background systems. However, it is known that *ex vivo* and *in vivo* tissues do not behave exactly the same because of the physiological changes which occur to *ex vivo* tissues when they are no longer part of a living body. As shown in Fig. 7(a), we compare an average spectrum of *ex vivo* breast cancer tumor xenograft to the *in vivo* tissue spectra. The differences, particularly between the *ex vivo* spectrum and its *in vivo* tumor counterpart, are appreciable. The absorption peaks due to water and to a lesser extent hemoglobin are less intense in the *ex vivo* spectrum—likely due to loss of blood flow, degree of oxygenation, and water in the sample. These observations suggest that in order to develop robust automated detections intraoperatively, *in vivo* measurements, specifically MCI measurements in humans, are critical.

## 4 Conclusions and Next Directions

*Ex vivo* feasibility and exploratory *in vivo* MCI studies have provided the groundwork for advancement of Vis-NIR MCI for real-time intraoperative applications. While *ex vivo* human renal tumor detection performance shows significant promise, the signatures measured do not necessarily represent signatures to be exploited from *in vivo* tissues. As a result, *in vivo* human studies, under IRB approval, are essential for method development. These studies will be undertaken using existing real-time MCI endoscopes under open, laparoscopic, and robot-assisted surgeries. In the future, as development of next generation handheld MCI devices become available, minimally invasive human surgeries will be undertaken to apply MCI for advanced surgical visualization.

## References

[1] Siegel R. L. Miller K. D. Jemal A., “Cancer statistics, 2019,” CA Cancer J. Clin. 69(1), 7–34 (2019). CAMCAM 0007-9235 10.3322/caac.21551 30620402

[2] The American Cancer Society, “Key statistics about kidney cancer,” 1 4, 2018, https://www.cancer.org/cancer/kidney-cancer/about/key-statistics.html.

[3] Garfield K. LaGrange C. A., “Cancer, renal cell,” StatPearls, 4 June 4, 2019, https://www.ncbi.nlm.nih.gov/books/NBK470336/ (accessed 12 1, 2019).

[4] King S. C. et al., “Continued increase in incidence of renal cell carcinoma, especially in young patients and high-grade disease: United States 2001–2010,” J. Urol. 191(6), 1665–1670 (2014). 10.1016/j.juro.2013.12.046 24423441 PMC4479175

[5] “Types of surgery for cancer treatment,” https://stanfordhealthcare.org/medical-treatments/c/cancer-surgery/types.html (accessed 1 12 2019).

[6] Mayo Clinic, “Cancer surgery: physically removing cancer,” 7 9, 2019, https://www.mayoclinic.org/diseases-conditions/cancer/in-depth/cancer-surgery/art-20044171 (accessed 1 12 2019).

[7] Volpe A. et al., “Contemporary management of small renal masses,” Eur. Urol. 60(3), 501–515 (2011). EUURAV 0302-2838 10.1016/j.eururo.2011.05.044 21664040

[8] Orosco R. K. et al., “Positive surgical margins in the 10 most common solid cancers,” Sci. Rep. 8, 5686 (2018). SRCEC3 2045-2322 10.1038/s41598-018-23403-5 29632347 PMC5890246

[r9] Borghesi M. et al., “Positive surgical margins after nephron-sparing surgery for renal cell carcinoma: incidence, clinical impact, and management,” Clin. Genitourin. Cancer 11(1), 5–9 (2013). 10.1016/j.clgc.2012.09.010 23083800

[r10] Ani I. et al., “Prevalence and impact on survival of positive surgical margins in partial nephrectomy for renal cell carcinoma: a population-based study,” BJU Int. 111(8), E300–E305 (2013). BJINFO 1464-410X 10.1111/j.1464-410X.2012.11675.x 23305148

[r11] Marszalek M. et al., “Positive surgical margins after nephron-sparing surgery,” Eur. Urol. 61(4), 757–763 (2012). EUURAV 0302-2838 10.1016/j.eururo.2011.11.028 22136987

[r12] Kwon E. O. et al., “Impact of positive surgical margins in patients undergoing partial nephrectomy for renal cortical tumors,” BJU Int. 99(2), 286–289 (2007). BJINFO 1464-410X 10.1111/j.1464-410X.2006.06623.x 17155985

[r13] Khalifeh A. et al., “Positive surgical margins in robot-assisted partial nephrectomy: a multi-institutional analysis of oncological outcomes (leave no tumor behind),” J. Urol. 190(5), 1674–1679 (2013). 10.1016/j.juro.2013.05.110 23764077

[14] Bansal R. K. et al., “Positive surgical margins during partial nephrectomy for renal cell carcinoma: results from Canadian Kidney Cancer information system (CKCis) collaborative,” Can. Urol. Assoc. J. 11(6), 182–187 (2017). 10.5489/cuaj.4264 28652876 PMC5472463

[15] Ryan S. et al., “Positive surgical margins after radical nephrectomy for localized kidney cancer: impact on survival,” J. Clin. Oncol. 37(7_Suppl.), 676–676 (2019). JCONDN 0732-183X 10.1200/JCO.2019.37.7_suppl.676

[16] Eldefrawy A. et al., “Negative impact of positive margins in partial nephrectomy in stage 1 renal cell carcinoma: a multicenter analysis,” in Am. Urol. Assoc. Annu. Meeting, Chicago (2019).

[17] Desmonts A. et al., “A new technique for ensuring negative surgical margins during partial nephrectomy: the *ex vivo* ultrasound control,” Prog. Urol. 23(12), 966–970 (2013). 10.1016/j.purol.2013.05.002 24090781

[r18] Doerfler A. et al., “*Ex vivo* ultrasound control of resection margins during partial nephrectomy,” J. Urol. 186, 2188–2193 (2011). 10.1016/j.juro.2011.07.100 22014810

[r19] Doerfler A. Oitchayomi A. Tillou X., “A simple method for ensuring resection margins during laparoscopic partial nephrectomy: the intracorporeal ultrasonography,” Urology 84, 1240–1242 (2014). 10.1016/j.urology.2014.07.025 25239259

[20] Veeratterapillay R. et al., “Intraoperative and surgical specimen (*ex vivo*) ultrasound in the assessment of margins at partial nephrectomy,” Int. Urol. Nephrol. 47, 1665–1669 (2015). IURNAE 0301-1623 10.1007/s11255-015-1083-0 26267670

[21] Hekman M. C. H. et al., “Intraoperative imaging techniques to support complete tumor resection in partial nephrectomy,” Eur. Urol. Focus 4(6), 960–968 (2018). 10.1016/j.euf.2017.04.008 28753888

[r22] Hoda M. R. Popken G., “Surgical outcomes of fluorescence-guided laparoscopic partial nephrectomy using 5-aminolevulinic acid induced protoporphyrin IX,” J. Surg. Res. 154, 220–225 (2009). JSGRA2 0022-4804 10.1016/j.jss.2008.12.027 19375717

[r23] Mitsui Y. et al., “Indocyanine green (ICG)-based fluorescence navigation system for discrimination of kidney cancer from normal parenchyma: application during partial nephrectomy,” Int. Urol. Nephrol. 44, 753–759 (2012). IURNAE 0301-1623 10.1007/s11255-011-0120-x 22215306

[24] Tobis S. et al., “Robot-assisted and laparoscopic partial nephrectomy with near infrared fluorescence imaging,” J. Endourol. 26, 797–802 (2012). 10.1089/end.2011.0604 22250958

[25] Altamar H. O. et al., “Kidney deformation and intraprocedural registration: a study of elements of image-guided kidney surgery,” J. Endourol. 25, 511–517 (2011). 10.1089/end.2010.0249 21142942

[26] Hughes-Hallett A. et al., “Augmented reality partial nephrectomy: examining the current status and future perspectives,” Urology 83, 266–273 (2014). 10.1016/j.urology.2013.08.049 24149104

[27] Treado P. et al., “Non-invasive and minimally invasive strategies for optical detection of cancer and anatomic structures,” in SPIE Bench-To-Bedside Conf., Unpublished (2015).

[28] Khan M. J. et al., “Modern trends in hyperspectral image analysis: a review,” IEEE Access 6, 14118–14129 (2018). 10.1109/ACCESS.2018.2812999

[29] Volynskaya Z. et al., “Diagnosing breast cancer using diffuse reflectance spectroscopy and intrinsic fluorescence spectroscopy,” J. Biomed. Opt. 13(2), 024012 (2008). JBOPFO 1083-3668 10.1117/1.2909672 18465975

[30] Kho E. et al., “Hyperspectral imaging for resection margin assessment during cancer surgery,” Clin. Cancer Res. 25(12), 3572–3580 (2019). 10.1158/1078-0432.CCR-18-2089 30885938

[31] Garcia-Uribe A. et al., “*In vivo* diagnosis of melanoma and nonmelanoma skin cancer using oblique incidence diffuse reflectance spectrometry,” Cancer Res. 72(11), 2738–2745 (2012). 10.1158/0008-5472.CAN-11-4027 22491533 PMC3367032

[32] Fawzy Y. S. et al., “*In vivo* assessment and evaluation of lung tissue morphologic and physiological changes from non-contact endoscopic reflectance spectroscopy for improving lung cancer detection,” J. Biomed. Opt. 11(4), 044003 (2006). JBOPFO 1083-3668 10.1117/1.2337529 16965160

[33] de Veld D. C. et al., “Autofluorescence and diffuse reflectance spectroscopy for oral oncology,” Lasers Surg. Med. 36(5), 356–364 (2005). LSMEDI 0196-8092 10.1002/lsm.20122 15856507

[34] Halicek M. et al., “Cancer detection using hyperspectral imaging and evaluation of the superficial tumor margin variance with depth,” Proc SPIE 10951, 109511A (2019). 10.1117/12.2512985 PMC7265739 32489227

[35] Evers D. J. et al., “Optical sensing for tumor detection in the liver,” Eur. J. Surg. Oncol. 39, 68–75 (2013). 10.1016/j.ejso.2012.08.005 22963834

[36] Bensalah K. et al., “Optical reflectance spectroscopy to differentiate benign from malignant renal tumors at surgery,” Urology 73, 178–181 (2009). 10.1016/j.urology.2008.08.462 18845323

[r37] Parekh D. J. Lin W. C. Herrell S. D., “Optical spectroscopy characteristics can differentiate benign and malignant renal tissues: a potentially useful modality,” J. Urol. 174(5), 1754–1758 (2005). 10.1097/01.ju.0000177484.33596.c9 16217277

[38] Couapel J.-P. et al., “Optical spectroscopy techniques can accurately distinguish benign and malignant renal tumors,” BJU Int. 111(6), 865–871 (2013). BJINFO 1464-410X 10.1111/j.1464-410X.2012.11369.x 22934843

[39] Krafft C. Popp J., “Micro-Raman spectroscopy in medicine,” Phys. Sci. Rev. 4(10), 1–15 (2019). 10.1515/psr-2017-0047

[40] Lu G. Fei B., “Medical hyperspectral imaging: a review,” J. Biomed. Opt. 19(1), 010901 (2014). JBOPFO 1083-3668 10.1117/1.JBO.19.1.010901 PMC3895860 24441941

[41] Wilson R. H. et al., “Review of short-wave infrared spectroscopy and imaging methods for biological tissue characterization,” J. Biomed. Opt. 20(3), 030901 (2015). JBOPFO 1083-3668 10.1117/1.JBO.20.3.030901 25803186 PMC4370890

[42] Bai C., “Noninvasive near infrared spectroscopy on living tissue with multivariate calibration approaches,” Dissertation, University of Iowa (2010).

[43] Ferrari M. et al., “Principles, techniques, and limitations of near infrared spectroscopy,” Can. J. Appl. Physiol. 29(4), 463–487 (2004). 10.1139/h04-031 15328595

[44] O’Sullivan T. D. et al., “Diffuse optical imaging using spatially and temporally modulated light,” J. Biomed. Opt. 17(7), 071311 (2012). JBOPFO 1083-3668 10.1117/1.JBO.17.7.071311 22894472 PMC3607494

[45] Nguyen J. Q. et al., “Spatial frequency domain imaging of burn wounds in a preclinical model of graded burn severity,” J. Biomed. Opt. 18(6), 066010 (2013). JBOPFO 1083-3668 10.1117/1.JBO.18.6.066010 PMC3680730 23764696

[46] Martens H. Naes T., Multivariate Calibration, John Wiley & Sons, New York (1989).

[47] Barker M. Rayens W., “Partial least squares for discrimination,” J. Chemom. 17(3), 166–173 (2003). JOCHEU 0886-9383 10.1002/cem.785

[48] Fawcett T., “An introduction to ROC analysis,” Pattern Recognit. Lett. 27(8), 861–874 (2006). PRLEDG 0167-8655 10.1016/j.patrec.2005.10.010

[49] Chan T. F. Vese L. A., “Active contours without edges,” IEEE Trans. Image Process. 10(2), 266–277 (2001). IIPRE4 1057-7149 10.1109/83.902291 18249617

[50] Rahunathan S. et al., “Image registration using rigid registration and maximization of mutual information,” in 13th Annu. Med. Meets Virtual Reality Conf., Long Beach, California (2005).

[51] Kosub S., “A note on the triangle inequality for the Jaccard distance,” Pattern Recognit. Lett. 120, 36–38 (2019). PRLEDG 0167-8655 10.1016/j.patrec.2018.12.007

[52] Koul H. et al., “Molecular aspects of renal cell carcinoma: a review,” Am. J. Cancer Res. 1(2), 240–254 (2011). 21969126 PMC3180049

[53] Couvidat C. et al., “Renal papillary carcinoma: CT and MRI features,” Diagn. Interventional Imaging 95(11), 1055–1063 (2014). 10.1016/j.diii.2014.03.013 25443332

